# High-Flow Nasal Cannula and Mandibular Advancement Bite Block Decrease Hypoxic Events during Sedative Esophagogastroduodenoscopy: A Randomized Clinical Trial

**DOI:** 10.1155/2019/4206795

**Published:** 2019-07-16

**Authors:** Wei-Nung Teng, Chien‐Kun Ting, Yu-Tzu Wang, Ming-Chih Hou, Wen-Kuei Chang, Mei-Yung Tsou, Huihua Chiang, Chun-Li Lin

**Affiliations:** ^1^Department of Anaesthesiology, Taipei Veterans General Hospital, Taipei City 11217, Taiwan; ^2^Department of Biomedical Engineering, National Yang-Ming University, Taipei City 11221, Taiwan; ^3^Department of Medicine, Taipei Veterans General Hospital, Taipei City 11217, Taiwan

## Abstract

During sedated endoscopic examinations, upper airway obstruction occurs. Nasal breathing often shifts to oral breathing during open mouth esophagogastroduodenoscopy (EGD). High-flow nasal cannula (HFNC) which delivers humidified 100% oxygen at 30 L min^−1^ may prevent hypoxemia. A mandibular advancement (MA) bite block with oxygen inlet directed to both mouth and nose may prevent airway obstruction during sedated EGD. The purpose of this study was to evaluate the efficacy of these airway devices versus standard management. One hundred and eighty-nine patients were assessed for eligibility. One hundred and fifty-three were enrolled. This study randomly assigned eligible patients to three arms: the standard bite block and standard nasal cannula, HFNC, and MA bite block groups. EGD was performed after anaesthetic induction. The primary endpoint was the oxygen desaturation area under curve at 90% (AUC_Desat_). The secondary endpoints were percentage of patients with hypoxic, upper airway obstruction, and apnoeic and rescue events. One hundred and fifty-three patients were enrolled. AUCdesat was significantly lower for HFNC and MA bite blocks versus the standard management (p= 0.019). The HFNC reduced hypoxic events by 18% despite similar airway obstruction and apnoeic events as standard group. The MA bite block reduced hypoxic events by 12% and airway obstructions by 32%. The HFNC and MA groups both showed a 16% and 14% reduction in the number of patients who received rescue intervention, respectively, compared to the standard group. The HFNC and MA bite block may both reduce degree and duration of hypoxemia. HFNC may decrease hypoxemic events while maintaining nasal patency is crucial during sedative EGD. The MA bite block may prevent airway obstruction and decrease the need for rescue intervention.

## 1. Introduction

The safety of sedation during esophagogastroduodenostomy (EGD) has long been a concern [1–3]. At least half of endoscopic procedures today are performed under monitored anaesthesia targeting deep sedation [4, 5]. The goal of sedation during endoscopy is to reduce patient pain and anxiety and ultimately provide better quality examination and follow-up [6]. Sedation using common medications such as midazolam, opioids, and propofol causes respiratory depression, hypoventilation, and subsequent hypoxemia [7]. Airway management is crucial during deep sedation, since desaturation is noted in up to 60% of patients [8] and as high as 95% of patients have different degrees of airway obstruction [9]. During deep sedation, the laryngeal muscle loses tone, causing partial or complete upper airway obstruction [10]. The breathing pattern also changes from mainly nasal breathing of an awake patient to mainly oral breathing of a mouth-open sedated patient [11]. Trained personnel manage the airway by chin-lift, jaw thrust, insertion of nasal airway, or bag-mask ventilation [12]. Serious complications such as cardiopulmonary distress, hypotension, bradycardia, or the need for intubation may occur if the airway is not properly treated [13].

High-flow nasal cannula (HFNC) is a device that delivers 10 to 70 L min^−1^ of heated, humidified 100% oxygen via nasal route. It provides positive airway pressure, decreases dyspnoea, decreases the work of breathing, and improves comfort. Studies have shown that it improves oxygenation in various perioperative settings such as the apnoeic patients, patients with respiratory failure [14], OSAS [15], and patients undergoing bronchoscopy [16]. The application of HFNC has been shown to decrease need for general anaesthesia in upper gastrointestinal procedures such as endoscopic retrograde cholangiopancreatography and endoscopic ultrasound [17]. However, the degree of oxygenation improvement in EGD has not been fully explored.

Mandibular advancement device (MAD) is a noninvasive alternative treatment for obstructive sleep apnoea syndrome (OSAS). It is recommended for patients with mild or moderate OSAS [18, 19]. The MAD mimics the jaw thrust action by holding the mandible in a forward position using splints to hold the upper and lower incisors in place to enlarge the upper airway [20]. The MA bite block is a device derived from MAD and designed for endoscopy performed via oral route. It provides mandibular advancement, supplemental oxygen directed to both nasal and oral cavities, and inlet for endoscopic entry. In our previous bench study comparing eleven airway devices for EGD, the MA bite block was the most capable device to deliver a high fraction of inspired oxygen (FiO_2_) [21]. Its clinical use has not been reported.

This study aimed to evaluate the role of HFNC and MA bite block during sedative EGD. The hypothesis was the use of these airway devices decrease duration and degree of hypoxic events compared with standard management devices.

## 2. Methods

### 2.1. Study Design

This study was a single centre multi arm parallel randomized clinical trial that compared high-flow nasal cannula (Optiflow, Fisher & Paykel, New Zealand) and MA bite block (Mandibular advancement bite block, Yong-Xu, Taiwan) against a standard bite block (MB-142 Reusable bite blocks, Olympus, Japan) and nasal cannula (Adult nasal cannula, Flexicare Medical Limited, United Kingdom). The three arms were standard bite block with standard nasal cannula, standard bite block with HFNC, and MA bite block. This study was approved by the Institutional Review Board of Taipei Veterans General Hospital, Taipei City, Taiwan (IRB #2017-03-003B), and written informed consent was obtained from all subjects participating in the trial. The trial was registered prior to patient enrolment at clinicaltrials.gov (NCT03138850, Principal investigator: Wei-Nung Teng, Date of registration: May 3, 2017). This manuscript adheres to the applicable CONSORT guidelines.

A computer-generated list of random numbers was used to allocate participants to one of three parallel groups in 1:1:1 ratio. Male and female patients aged 20 to 80 years with American Society of Anaesthesiologists (ASA) physical status I to II who were undergoing routine outpatient EGD were eligible for enrolment. The exclusion criteria included allergy to the study medications, poor incisor teeth stability, anticipated procedure duration of greater than 30 minutes, history of gastroparesis, history of facial or oral surgery, and baseline oxygen saturation <90% on room air. The study took place at the Taipei Veterans General Hospital Endoscopy Centre for Diagnosis and Treatment, Taipei City, Taiwan. Written informed consent was obtained from all participants. Follow-up for all patients concluded on day of recruitment. Before the start of examination, a study nurse conducted the STOP-Bang questionnaire for risk of OSAS [22]. It consisted of eight yes/no questions scored 1 or 0, and thus the highest score was 8 and lowest 0. A yes to 0-2, 3-4, or 5 and above presented low, moderate, and high risks for OSAS, respectively.

**Figure 1:**
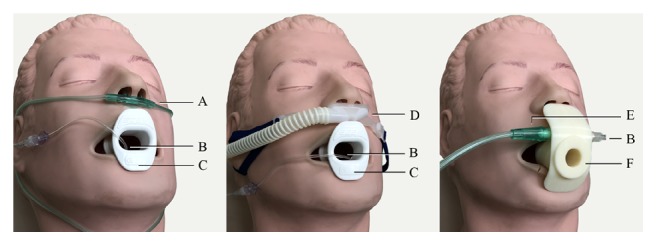
Study set-up. Left: standard bite block group; middle: HFNC group; right: MA bite block group. HFNC: high-flow nasal cannula; MA: mandibular advancement; A: nasal cannula; B: capnography catheter; C: standard bite block; D: high-flow nasal cannula; E: oxygen cannula; F: mandibular advancement bite block.

Standard anaesthetic monitors including electrocardiogram, noninvasive blood pressure, and oxygen saturation via pulse oximeter were applied to each patient. Bispectral index (BIS) monitor was also applied to allow objective evaluation of the level of sedation achieved and prevent the adverse events of overdose [23]. The patients were asked to position themselves into left decubitus position. Five L min^−1^ oxygen was delivered to the standard bite block group via a standard nasal cannula. For HFNC group, 100% oxygen was given at flow rate of 30 L min^−1^ [24]. An oral catheter detected end-tidal carbon dioxide (ETCO_2_). The MA bite block was equipped with connectors for oxygen inlet directed to both nose and mouth and ETCO_2_ detection catheter. ETCO_2_ detection and 5 L min^−1^ oxygen were connected to the bite block in the MA bite block group [21] (Figure 1). Once the bite blocks were placed and the patients were preoxygenated for 5 minutes, midazolam intravenous bolus was administered at a dose of 0.05 mg kg^−1^, and alfentanil at 0.2 mcg kg^−1^. No additional midazolam or alfentanil was permitted for the remainder of the procedure. Propofol was initiated via target controlled infusion (TCI) (Agilia, SB Medica SRL, Italy) at 1 mcg ml^−1^ and was titrated throughout the procedure by the in charge anaesthesiologist to maintain moderate to deep sedation [25]. The Modified Observer's Assessment of Alertness/Sedation (MOAA/S) score was assessed every 30 seconds by the anaesthesiologist in charge, beginning after initial drug dose administration and continuing until the patient recovered. Immediately after the anaesthesiologist confirmed that the patients reached a MOAA/S score of < 2 (responds only after mild prodding or shaking), the EGD was initiated by the endoscopist. The time of examination was defined as time of endoscopic insertion to removal of endoscopy. Rescue intervention such as sustained chin-lift or jaw thrust, insertion of an oral or nasal airway, or bag-mask ventilation was done and recorded by anaesthesiologist in charge.

A data acquisition system (Datex/Omeda S/5 Collect, GE Corporate, USA) was used to electronically collect data every 10 seconds on all physiologic measurements such as heart rate, none invasive blood pressure, oxygen saturation, ETCO_2_, impedance, BIS, etc. After data collection, a separate anaesthesiologist blinded to the allocation groups evaluated the endpoints.

### 2.2. Endpoints

The primary endpoint was the area under the curve of 90% oxygen desaturation (AUC_Desat_) [26, 27]. The AUC_Desat_ was defined as the integrated area under oxygen saturation (SpO_2_) for a selected cut point per 10 seconds. For example, if a person's oxygen saturation was 85% for a period of 30 seconds, the AUC_Desat_90% for that duration was 150 (seconds %). The AUC_Desat_ combined the incidence, duration, and magnitude of a patient's oxygen desaturation and better reflected the duration and severity of hypoxemia than the lowest saturation or the number of hypoxic events [28, 29].

Secondary endpoints were the number of patients with airway obstruction episodes, apnoea episodes, who received rescue interventions, and hypoxic events. Grunting or snoring with positive ETCO_2_ measurement was defined as partial airway obstruction episodes. Loss of ETCO_2_ detection while in presence of respiratory activity was defined as complete airway obstruction. Lack of respiratory activity and loss of ETCO_2_ detection greater for 30 seconds were defined as apnoea episodes. Hypoxic event was defined as number of patients experiencing episodes of oxygen saturation < 90%.

### 2.3. Statistical Analyses

The sample size calculation was based on ratio of hypoxic events due to the fact that the primary endpoint, AUC_Desat_, is a new measure in procedural sedation, and as a result, a sample size could not be calculated from published AUC_Desat_ data. From our preliminary study, the ratio of hypoxic events in the standard vs. intervention group was 25% vs. 5%. The sample size was calculated by anticipated incidence comparison of independent sample study and was estimated to be 50 patients in each group to achieve a power of 0.8 and *α* = 0.05. Continuous variables were compared with analysis of variance, with Tukey H test as post hoc to determine demographic comparability. The Chi-square test compared categorical variables including patient characteristic and secondary endpoints. The primary analysis was a between-group comparison of AUC_Desat_, performed using the Kruskal-Wallis H test and Dunn test post hoc. SPSS 24.0 (SPSS, Inc., Chicago, IL, USA) performed all statistical analysis.

## 3. Results

**Figure 2:**
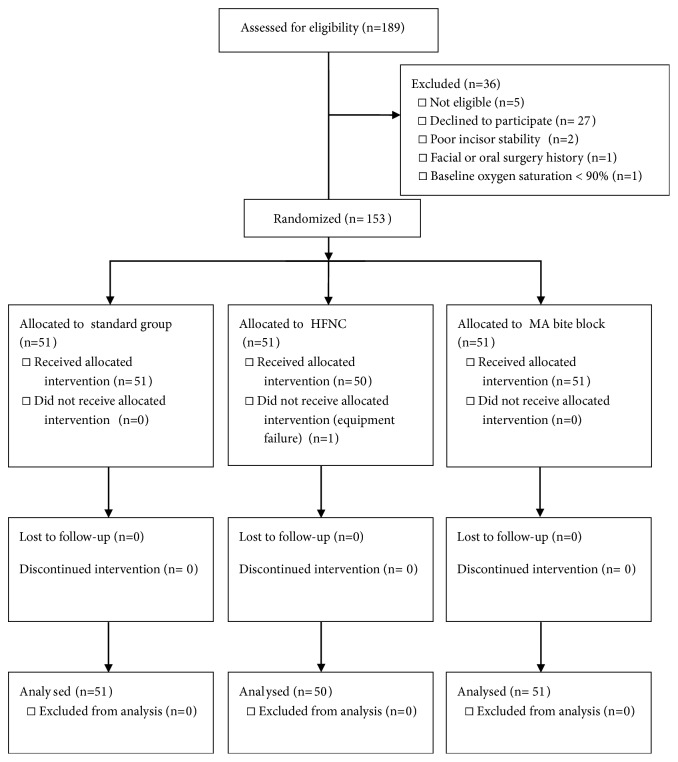
CONSORT flow diagram. HFNC: high-flow nasal cannula; MA: mandibular advancement.

One hundred and eighty-nine patients were assessed for eligibility from May 3rd to June 22nd, 2017. The flow diagram was illustrated in Figure 2. Ten anaesthesiologists and five endoscopists participated in this study. Patient characteristics data showed no differences in sex, gender, weight, height, and body mass index (BMI) between groups (Table 1). There were significantly more ASA class II participants in HFNC group. The risk for OSAS by STOP-Bang score also showed no significant differences between groups.

Exam profile showed no differences in BIS index, averaging in the light sedation range of 75~78 in all groups. There was no difference between time from end of preoxygenation to when patient reach MOAA/S < 2 and total exam time between the three groups. The effective site TCI propofol concentration was significantly higher in HFNC group (p=0.001) (Table 1).

**Figure 3:**
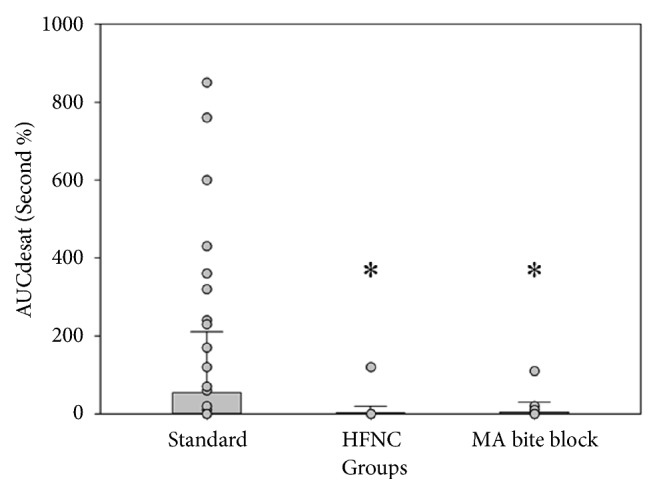
Primary outcome: bar plot of AUC_Desat_. AUC_Desat_: area under the curve for oxygen saturation below 90%; HFNC: high-flow nasal cannula group; MA bite block: mandibular advancement bite block group; *∗*: p < 0.05.

In primary endpoint analysis, there were significant reductions in the HFNC and MA groups (mean AUC_Desat_ = 5.22 sec %, 3.26 sec %, respectively), whereas the mean AUC_Desat_ of standard group was 54.37 sec % (p = 0.019) (Figure 3).

For secondary endpoints, percentage of patients experiencing partial and total airway obstructions did not differ for the HFNC group from standard group (62% vs. 63%). The percentage was reduced by 32% in the MA group.

The number of apnoea episodes did not differ between standard and intervention groups. The HFNC and MA groups both showed a 16% and 14% reduction in the number of patients who received rescue intervention respectively compared to the standard group. For number of patients experiencing hypoxic events, there were 18% reduction in the HFNC and 12% in MA groups vs. standard group (Table 2).

Patients using the HFNC and MA bite block did not complain of any nasal, teeth, gum, or jaw discomfort. Endoscopists did not find interference with EGD entry or manoeuvring. However, in the standard bite block group, two patients reported nose dryness and itching from oxygen via nasal cannula. No serious complications such as hypotension, cardiopulmonary distress, hypoxemia requiring bag-mask ventilation, or intubation occurred in this study.

## 4. Discussion

This study showed that both the HFNC and MA bite blocks significantly reduced the degree and duration of hypoxic events. There was significantly less AUC_Desat_ in the HFNC group despite similar airway obstruction and apnoeic event ratio. The MA group demonstrated significantly less AUC_Desat_, while decreasing airway obstruction and hypoxic events. The percentage of patients using the standard bite block with airway obstruction and hypoxic events was compatible with previous studies of 60~95% and 47~80%, respectively [8, 9, 30].

One patient experienced hypoxic event out of fifty in the HFNC group. Although number of apnoeic or airway obstruction events were not statistically different from standard group, application of HFNC still showed satisfactory results. This may be due to the 100% oxygen delivery of HFNC in this study, when using HFNC at similar FiO_2_ as standard nasal cannulas may not prevent desaturation in high risk patients [31]. While HFNC provides excellent oxygenation and prolongs the apnoeic period, maintaining airway patency is crucial [15]. The HFNC did not guarantee total prevention of hypoxemia in this study. This may be due to frequent apnoeic events during sedative EGD. The aim of high-flow oxygen therapy is to meet patient's peak inspiratory flow rate [24]; however when a patient is apnoeic, the flow of 30 L min^−1^ may not be sufficient to prevent hypoxia events. Nasal route may be obstructed during sedative EGD. We have found that breathing was conducted primarily via oral breathing in 58% of patients under sedation in a previous study [11]. Under EGD, the percentage of oral breathing was further increased with oral capnography capturing 100% of patients [11]. Since HFNC is given via nasal route, maintaining nasal patency would be an important factor in oxygen delivery. The apnoeic and airway obstruction events were analysed via an oral ETCO_2_ catheter, by which nasal obstruction may not be detected. No nasal airway devices were used in this study. However, ensuring nasal patency may improve efficacy when using HFNC during sedative EGD.

The MAD is derived from the jaw thrust manoeuvre. The device consists of upper teeth and lower teeth splints that protrude the mandible via the genioglossus muscles. The MAD is reported to be effective in 28~80% of OSAS patients [18, 19, 32]. One reason for partial treatment may be the etiology of the obstructed airway. Sasao et al. [33] evaluated airway morphology using nasopharyngoscopy and demonstrated that, in severe OSAS patients, the MAD widened oro-hypopharynx in 100% of patients but widened the velopharynx by only 81%. As a result, apnoea-hypopnea index was reduced by only 40.6% in those with partial widening. Although there is no effective model to predict patients suitable for MAD, many predictors of successful treatment are suggested, including supine-dependent apnoea, mild sleep apnoea, degree of mandibular advancement, and female gender [34, 35]. The ratio of risks for OSAS and gender in this study was the same between groups. Therefore, these alone may not explain the decrease in hypoxemia.

In a conscious patient, breathing is conducted primarily via the nostrils. When sedated, breathing pattern changes to oral breathing in 58% of patients. Under EGD, the percentage of oral breathing is further increased with oral capnography capturing 100% of patients [11]. Oxygen supplementation to both nasal and oral routes is equally important during sedative EGD. The MA bite block in this study is equipped with an oxygen inlet directing oxygen flow to both nasal and oral routes, increasing oxygen delivery efficacy. In our previous bench study, when given oxygen flow of 5 L min^−1^, the MA bite block delivered 63% FiO2 during normoventilation and a high 80% during hypoventilation [21]. During hypoventilation, FiO_2_ increases to 86%. Comparing those results to capability of oxygen delivery of nasal cannulas of 40% at oxygen flow of 5 L min^−1^, the MA bite block may be more capable of sustaining a high FiO2. Its high oxygen delivery capability may also contribute to the decrease in hypoxemia.

There were several limitations in this study. Some of the patients eligible for this study refused to participate. The reason for refusal may be due to lack of understanding of the newer devices and fear of deviation from standard protocol. To minimize bias, patients were randomized by computer-generated protocol and the anaesthesiologist in charge was not involved in patient selection and data analysis. Further, patients were excluded from this study based on predetermined criteria before enrolment. Small sample size is another limitation. Therefore, recalculation of sample size based on results of AUC_Desat_ may be needed for further studies. Furthermore, the results of this study only apply to the Asian population with normal BMI and normal incisor status. The application of the HFNC and MA bite block to high risk patients such as obesity, severe OSAS, or limited pulmonary function needs further investigation.

## 5. Conclusions

This study showed promising improvement in oxygenation and decreases in rescue intervention with the HFNC and MA bite block during sedative endoscopy. HFNC at 30 L min^−1^ may provide sufficient oxygenation; however maintaining nasal patency is crucial to prevent hypoxemia. Examination safety may be improved by using a simple device that enables endoscope entry as well as maintaining a patent airway. Further study is required to determine efficacy of the HFNC and MA bite block in high risk patients.

## Figures and Tables

**Table 1:** Patient and endoscopic exam characteristics.

Groups new	standard (n=51)	HFNC (n=50)	MA bite block (n=51)	*p*
Age (years)	51.56 ± 12.52	46.65 ± 15.37	51.07 ± 11.96	0.130
Sex (Male *n*/ Female *n*)	22 / 29	19 / 31	19 / 32	0.804
ASA (I *n*/ II *n*)	22 / 29	7 / 43	20 / 31	0.003*∗*
Weight (kg)	63.89 ± 11.85	62.46 ± 13.20	61.26 ± 14.56	0.605
Height (cm)	162.57 ± 8.84	163.44 ± 6.74	161.61 ± 8.30	0.522
BMI (kg/m^2^)	23.44 ± 3.58	22.51 ± 4.19	22.90 ± 3.58	0.466
STOP-Bang risk (low *n*/mod *n*/high *n*)	21/17/13	26/13/11	32/12/6	0.208

Mean time to reach MOAA/S < 2	1.77 ± 0.76	2.12 ± 1.54	2.04 ± 1.02	0.279
Exam time	5.83 ± 2.33	5.96 ± 1.69	6.47 ± 2.12	0.263
BIS	75.29 ± 14.27	78.08 ± 14.84	75.66 ± 16.99	0.616
Propofol TCI Ce	1.05 ± 0.41	1.45 ± 0.51	1.29 ± 0.59	0.001*∗*

Data are represented as mean ± standard deviation.

ASA: American Society of Anesthesiologists classification, BMI: body mass index, STOP-Bang: STOP-Bang questionnaire; MOAA/S: Modified Observer's Assessment of Alertness/Sedation; BIS: Bispectral index; Propofol TCI Ce: effect site concentration for propofol target controlled infusion; HFNC: high-flow nasal cannula; MA: mandibular advancement.

**Table 2:** Secondary endpoints.

Groups	standard (n=51)	High-flow nasal cannula (n=50)	Mandibular advancement bite block (n=51)	*p*
Airway obstruction	32 (63)	31 (62)	16 (31)	<0.001*∗*
Partial obstruction [n (%)]	13 (26)	19 (38)	10 (20)	
Complete obstruction [n (%)]	19 (37)	20 (40)	6 (12)	
Apnoeic events [n (%)]	19 (37)	24 (38)	19 (29)	0.448
Hypoxic events [n (%)]	11 (20)	1 (2)	4 (8)	0.004*∗*
Rescue events [n (%)]	9 (18)	1 (2)	2 (4)	0.006*∗*

## Data Availability

The data used to support the findings of this study are available from the corresponding author upon request.
